# Cool birds: first evidence of energy-saving nocturnal torpor in free-living common swifts *Apus apus* resting in their nests

**DOI:** 10.1098/rsbl.2021.0675

**Published:** 2022-04-13

**Authors:** Arndt H. J. Wellbrock, Luca R. H. Eckhardt, Natalie A. Kelsey, Gerhard Heldmaier, Jan Rozman, Klaudia Witte

**Affiliations:** ^1^ Research Group of Ecology and Behavioural Biology, Institute of Biology, University of Siegen, Siegen, Germany; ^2^ Institute of Avian Research ‘Vogelwarte Helgoland’, Wilhelmshaven, Germany; ^3^ Animal Physiology, Faculty of Biology, Marburg University, Marburg, Germany; ^4^ Czech Centre for Phenogenomics, Institute of Molecular Genetics of the Czech Academy of Sciences, Vestec, Czech Republic

**Keywords:** nest temperature, metabolic rate, field study, CaloBox™, hypometabolism, non-invasive methods

## Abstract

Daily torpor is a means of saving energy by controlled lowering of the metabolic rate (MR) during resting, usually coupled with a decrease in body temperature. We studied nocturnal daily torpor under natural conditions in free-living common swifts *Apus apus* resting in their nests as a family using two non-invasive approaches. First, we monitored nest temperature (*T*_nest_) in up to 50 occupied nests per breeding season in 2010–2015. Drops in *T*_nest_ were the first indication of torpor. Among 16 673 observations, we detected 423 events of substantial drops in *T*_nest_ of on average 8.6°C. Second, we measured MR of the families inside nest-boxes prepared for calorimetric measurements during cold periods in the breeding seasons of 2017 and 2018. We measured oxygen consumption and carbon dioxide production using a mobile indirect respirometer and calculated the percentage reduction in MR. During six torpor events observed, MR was gradually reduced by on average 56% from the reference value followed by a decrease in *T*_nest_ of on average 7.6°C. By contrast, MR only decreased by about 33% on nights without torpor. Our field data gave an indication of daily torpor, which is used as a strategy for energy saving in free-living common swifts.

## 1. Introduction

Torpor is a highly controlled and reversible physiological state of hypometabolism [1,2] observed in many endotherms (i.e. birds and mammals) [3–6]. In birds, torpor usually lasts less than 24 h, called daily torpor [4]. The metabolic rate (MR) during daily torpor may be lowered by about 50% [7–9] up to 95% in hummingbirds [6]. MR lowering can be accompanied by a temporary decrease in body temperature (*T*_b_ by approximately 5–30°C [9,10]) depending on the ambient temperature [7].

Among other functions [11,12], daily torpor enables endotherms to cope with times of energetic stress due to food shortage and/or cold periods [13,14]. Many birds escape unfavourable environments by migration; hence, daily torpor can often be found in resident species like mousebirds and New Zealand wrens [15–18]. However, migratory birds can be confronted to energetic stress when staying at their breeding sites, leading to an occasional use of daily torpor [19,20]. This applies especially to bird species breeding in unpredictable environments with varying food availability [21], such as in insectivorous birds like nightjars [19,20,22] or swifts [23].

The common swift *Apus apus* often faces cold periods at their breeding sites, which span across Europe and beyond the Arctic Circle [24–26]. Anecdotally, it was reported that free-living breeding common swifts enter a nocturnal torpid state during harsh weather conditions which lower the activity of airborne insects for several days (personal observation by J.R., [27,28]). Previous laboratory studies in fasting common swifts kept in respirometry chambers showed that both juvenile (from 13 to 15 days of age) and adults can lower *T*_b_ and MR reversibly during resting in times of food and water deprivation at low ambient temperature (*T*_a_) [29–32]. However, there have been no systematic studies on the occurrence and frequency of nocturnal daily torpor in free-living common swifts in the wild during the short breeding season. Therefore, we studied families of common swifts resting at their nests at a German breeding site for 8 years. To minimize disturbance, we used two non-invasive methods: (i) we measured drops in nest temperature (*T*_nest_) with temperature loggers fixed inside nests in 2010–2015 and 2017–2018, a method validated for quantifying torpor [33] and nest attendance [34–37]. (ii) Since a substantial drop in *T*_nest_ is not sufficient to detect torpor [38], as shown, e.g. for *T*_b_ in tropical animals [39,40], we additionally measured MR of the families directly at their nests in 2017–2018.

Based on the high dependence on airborne food and therefore on weather, previous anecdotal evidence and former laboratory studies, we predict that daily torpor events (hereafter ‘torpor’), indicated by a lower MR accompanied by a *T*_nest_ drop, would regularly occur in families of resting common swifts during breeding seasons on nights with comparatively low *T*_a_.

## 2. Methods

A detailed description of the methods is given in the electronic supplementary material. Fieldwork was conducted at a common swift colony of 29–55 pairs breeding in natural open nests inside walk-in chambers of a concrete highway bridge (51°02′28″ N, 7°49′36″ E) near the city of Olpe, Germany, during breeding seasons in 2010–2015 and 2017–2018. Throughout each breeding season (end of April to early August), we embedded iButton™ temperature loggers (type DS1922 L; accuracy ± 0.5°C; Maxim Integrated™, USA) into the lower part of nest walls to measure *T*_nest_ at 5 min intervals (electronic supplementary material, figure S1). *T*_a_ inside the walk-in chambers was measured near the nests with a data logger. We analysed *T*_nest_ of 24–50 nests per breeding season that were continuously occupied during nights for at least 30 days (table 1). As an indicator of torpor [33], we counted substantial *T*_nest_ drops (i.e. a drop with a difference between *T*_nest_ and *T*_a_ ≤ 7°C, a criterion validated in a pilot study with video-taped nests using infrared video cameras and iButtons™ embedded in the nest). Thereby, the pattern of *T*_nest_ drops has to resemble the *T*_b_ profile during torpor cycles recorded in laboratory studies [29–32].

**Table 1.** Number of monitored nests observed for at least 30 days per breeding season, number of observations (no. nests × nights), number of nests with *T*_nest_ drops (difference between *T*_nest_ and *T*_a_ ≤ 7°C), number of nights with *T*_nest_ drops, percentage of nests with at least one *T*_nest_ drop and percentage of nights with *T*_nest_ drops.

year	no. observed nests	no. observations (no. nests × nights)	no. nests with *T*_nest_ drops	no. nights with *T*_nest_ drops	percentage of nests with at least one drop in *T*_nest_	percentage of nights with *T*_nest_ drops
2010	24	1507	11	24	46	1.6
2011	34	2181	20	25	59	1.1
2012	43	3078	38	82	88	2.7
2013	43	3149	43	187	100	5.9
2014	50	3393	32	61	64	1.8
2015	48	3365	29	44	60	1.3
2017	48	3131	29	46	60	1.5
2018	41	2553	11	16	27	0.6

In addition, in 2017 and 2018, seven pairs nested inside wooden boxes (40 × 20 × 20 cm, v. 16 l) equipped for metabolic measurements (electronic supplementary material, figure S2). We used one mobile indirect calorimetry system CaloBox™ (electronic supplementary material, figure S3) [41] to record oxygen consumption (${\dot{V}}_{O_{2}}$)and carbon dioxide production (${\dot{V}}_{{\mathrm{CO}}_{2}}$) of resting families up to six individuals.

We recorded gas exchange on 31 nights between 26 June and 28 July 2017 and the same number of nights from 13 June to 17 July 2018: three nests (nest IDs 142, 175, 183) with 1–4 nestlings (age 17–48 days) in 2017 and two nests (nest IDs 173, 175) with three eggs and 2–3 nestlings (age 0–27 days) in 2018 (electronic supplementary material, table S1). Since number of birds and total body mass in the nest are major determinants of ${\dot{V}}_{O_{2}}$, we controlled for the number of parents present, from none to both adults at the nest, on nine of the 62 nights. We know from a previous study in the same colony [42] and other sites [27,28], that adults do not leave (or enter) the nest during darkness. Hence, we assumed that the number of birds did not vary during the calorimetric measurements. Total mass of all birds during measurements ranged from approximately 47 g (one nestling, nest ID 175) to 239 g (four nestlings and two adults, nest ID 183; electronic supplementary material, table S2).

Following the general definitions of torpor in the literature, torpor is often characterized by an approximately 50–95% decrease in MR for individuals [8,9,43]. Although we are aware that we are measuring groups of birds and that there is no clear threshold for torpor events, we used a relative reduction in resting MR by approximately 50% in our study as a conservative guideline for torpor events, accompanied by a substantial *T*_nest_ drop [43]. We know from video monitoring that adults arrive near sunset at the nest and start resting a few minutes later after feeding the young. To be sure that we measured ${\dot{V}}_{O_{2}}$ during resting, we used the ${\dot{V}}_{O_{2}}$ value half an hour after ${\dot{V}}_{O_{2}}$ peak (triggered by the adults’ arrival) as a reference value (MR_ref_) to calculate relative MR reduction (%) based on the formula (MR_ref_ - MR_min_) × 100/MR_ref_ with MR_min_ being the lowest ${\dot{V}}_{O_{2}}$ value during the night. Based on MR reduction (greater or lower than approximately 50%), we separated the 62 nights with metabolic data into nights with and without torpor. As the initial value for drop in *T*_nest_, we used the value at the time from which *T*_nest_ decreased continuously after the arrival of the adults. Relationships between the parameters of *T*_nest_ and MR (e.g. duration, relation to sunset or sunrise) were assessed using Pearson's product-moment correlation. We compared absolute *T*_nest_ and relative ${\dot{V}}_{O_{2}}$ reduction on nights with torpor with an equal number of nights without torpor from the same nests. We assessed non-torpor nights which were either before or after nights with torpor events, based on weather forecasts and the time period of measurements at each nest (electronic supplementary material, table S1). Since *T*_a_ affects all nests equally, we included all days with complete MR measurements (total *n* = 60) in the comparisons of the daily averaged *T*_a_ during days previous to nights with torpor with the daily *T*_a_ during days previous to nights without torpor. For these comparisons, we applied linear mixed models (LMM, R-packages ‘lme4’ [44] and ‘sjPlot’ [45]). Year and nest ID were random factors. Model assumptions (e.g. normal distribution of residuals and Tukey–Anscombe plot) were assessed graphically following [46]. All data analyses were performed in R [47].

## 3. Results

In 2010–2015, we found 24 to 187 substantial drops in *T*_nest_ per breeding season (table 1; total of 423 events in 16 673 observations; 1.1–5.9% of examined nights per season) with at least one *T*_nest_ drop in 70 ± 20% (range: 46–100%) of occupied nests (range: 24–50 nests). On average, *T*_nest_ dropped by 8.6 ± 2.7°C (range: 3.0–18.0°C) and a *T*_nest_ drop lasted around 10.8 ± 3.3 h (range: 4–22 h, *n* = 423 events). In 2017 (48 nests) and 2018 (41 nests), we counted 46 and 16 *T*_nest_ drops, respectively (table 1; total of 62 events in 5684 observations; 1.5% and 0.6% of nights examined). Of this total of 62 *T*_nest_ drops, 29 and nine drops, respectively, were detected during the period of the MR measurements (31 nights per year).

**Figure 1.**
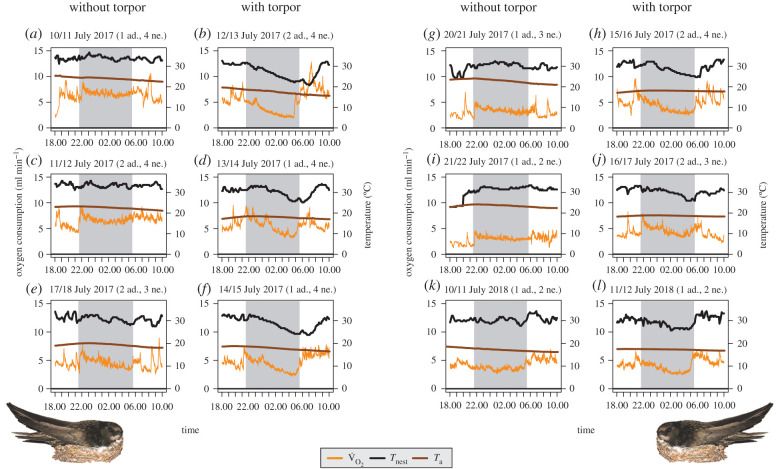
Oxygen consumption (${\dot{V}}_{O_{2}}$), nest temperature (*T*_nest_) and ambient temperature (*T*_a_) inside the bridge during six nights each without (*a,c,e,g,i,k)* and with torpor (*b,d,f,h,j,l)* in two nests (nest ID 183: *a–j*; nest ID 175: *k,l*). Area shaded grey indicates night. Number of individuals at the nest are given in brackets (ad. = adult; ne. = nestling).

The arrival of adults at the nests near sunset was evident from a steep increase in ${\dot{V}}_{O_{2}}$. We found a substantial decrease in ${\dot{V}}_{O_{2}}$ for five of the 31 nights in 2017 (nest ID 183, 3–4 nestlings and 1–2 adults, figure 1). In 2018, which was the warmest year since weather records began in Germany, we found a substantial decrease in ${\dot{V}}_{O_{2}}$ in one of the 31 nights (nest ID 175, two nestlings and one adult; figure 1). In these two nests (nest ID 183 and 175), MR decreased on average by 56 ± 6% of MR_ref_ (range: 49–62%) on the six nights where torpor was used, and MR reduction was 33 ± 9% (range: 23–46%) in the equal number of nights without torpor (*n* = 6; figure 1). ${\dot{V}}_{O_{2}}$ reduction on the six nights with torpor started 19 ± 42 min after sunset (range: 20 min before to 88 min after sunset) and ended 45 ± 35 min before sunrise (range: 7–102 min). The later the ${\dot{V}}_{O_{2}}$ reduction started after sunset, the shorter the bout duration (*r* = 0.84, *p* = 0.04). By contrast, there was no correlation between bout duration and the start of arousal relative to sunrise (*r* = −0.37, *p* = 0.47), meaning that short and long torpor bouts have a similar temporal distance to sunrise. The lowest ${\dot{V}}_{O_{2}}$ value measured was 94 ± 39 min (55–146 min) before sunrise.

On the six nights with a substantial decrease in ${\dot{V}}_{O_{2}}$ in 2017 and 2018, *T*_nest_ decreased on average by 7.6 ± 1.8°C with a mean lowest temperature of 24.3 ± 1.8°C (range: 21–26°C; figure 1). This drop was 3.6 ± 1.2°C on the six nights without torpor with a mean lowest temperature of 30.1 ± 1.8°C (range: 28–32°C). On nights with torpor, *T*_nest_ declined 81 ± 46 min (range: 11–130 min) after onset of ${\dot{V}}_{O_{2}}$ decrease. The lowest *T*_nest_ values on nights with torpor were negatively correlated with duration of *T*_nest_ drops (8.6 ± 1.9 h, range 6.0–11.2 h; *r* = −0.93, *p* < 0.01) but not with duration of MR reduction (7.6 ± 0.7 h, *r* = 0.07, *p* = 0.90). There was a positive correlation between absolute *T*_nest_ decrease and relative ${\dot{V}}_{O_{2}}$ decrease on the six torpor nights (*r* = 0.84, *p* = 0.04). Average *T*_a_ was significantly lower during days previous to nights with torpor than during days previous to nights without torpor (table 2).

**Table 2.** Ambient temperature (*T*_a_) inside the bridge (mean and confidence intervals CI of LMM) on days with or without torpor.

parameter	days with nocturnal torpor *n* = 6	CI	days without nocturnal torpor *n* = 54^a^	CI	*n*	*R*^2^	*p*-value
mean *T*_a_ [°C]	17.3	14.9–19.7	20.8	18.3–23.3	60^a^	0.138	0.005
min. *T*_a_ [°C]	16.2	13.8–18.6	19.5	17.2–21.8	60^a^	0.130	0.006
max. *T*_a_ [°C]	18.5	16.1–20.9	22.6	19.7–25.5	60^a^	0.183	0.001

^a^Incomplete *T*_a_ measurements by the CaloBox™ on the first and last day were excluded.

## 4. Discussion

We regularly detected substantial drops in *T*_nest_, indicating daily torpor in families of free-living common swift under natural conditions at a low frequency of 1–6% of nights within a season. Such drops in *T*_nest_ were found at least once in the majority of nests per season, except for 2018 (only 27% of nests). In 2017 and 2018, we detected six events of substantial ${\dot{V}}_{O_{2}}$ decrease by 56 ± 6% (relative to MR_ref_) in two nests with groups of up to six individuals accompanied by a *T*_nest_ drop during nights with comparatively low *T*_a_. MR reductions started shortly before or after sunset whereas increases were always initiated before sunrise. Given the magnitude of our chosen conservative threshold for torpor, i.e. MR reduction of about 50%, we conclude that the observed MR declines in free-living common swifts represent nocturnal torpor among some or all individuals resting together in a nest. As Willis *et al*. [33], we found time-lagged correlations between *T*_nest_- and MR-defined torpor entry, arousal onset and completion.

Laboratory studies in juvenile and adult common swifts under fasting conditions provided the only evidence of torpor in common swifts to date [29–32]. Koskimies [29,30] found that individuals' relative ${\dot{V}}_{O_{2}}$ decreased by approximately 39% and 69% in two juveniles and about 60% in an adult, which corresponds with our findings ranging from 49% to 62% for a group. Due to the nature of our field study, we could only record torpor events within a family or a breeding pair. The calculation of individual energy savings requires further technical equipment (thermal imaging cameras [48]) or invasive methods (implants to monitor heart rate [49]), which are hardly applicable in common swifts. Defining torpor for a group is complicated because individual members can differ in body mass, size, energy reserves, and thus, in the propensity to undergo torpor [44,50,51]. However, other bird species resting in a group are known to be highly synchronized in *T*_b_ and MR as shown e.g. in mousebirds [15,52,53] or bronze mannikins (*Spermestes cucullatus*) [54]. Moreover, social thermoregulation can facilitate and even enhance energy savings, e.g. of 50% in free-ranging white-backed mousebirds (*Colius colius*) [15]. Therefore, it is possible that individual torpor is masked by the higher MR of other family members [55]. In this case, we might underestimate the numbers of actual torpor events in our study.

To conclude, three open questions arise: (i) what supports the arousal from torpor, (ii) what is the adaptive value of torpor and (iii) what impact does torpor have on life-history decisions? Brown adipose tissue, used for non-shivering thermogenesis (NST) in eutherian mammals, has not yet been found in birds [56,57]. Therefore, muscle NST likely supports the arousal from torpor in birds [57–60] together with active muscle shivering and increasing heart rate, which has been observed in captive common swifts [32]. From these swifts, we know that fasting can induce torpor bouts, which become more pronounced with prolonged food deprivation. Therefore, we assume that food shortage is also the main cause for torpor in free-living common swifts similar to Alpine swifts (*Tachymarptis melba*) [23]. Since food availability, i.e. abundance of airborne insects, is reduced at low temperatures [61], it is expectable that we found indications for torpor rather rarely because cold weather is infrequent during the warm breeding season. We hypothesize that the ability to reduce MR helped common swifts to expand their breeding range into northern Palaearctic regions. Daily torpor might enable common swifts to cope with potentially increasing extreme weather events due to climate change [62].

## Data Availability

Data are available from the Dryad Digital Repository: https://doi.org/10.5061/dryad.6wwpzgn1f [63]. Further methodological details and field data are provided in the electronic supplementary material [64].

## References

[1] Heldmaier G, Ortmann S, Elvert R. 2004 Natural hypometabolism during hibernation and daily torpor in mammals. Respir. Physiol. Neurobiol. **141**, 317-329. (10.1016/j.resp.2004.03.014) 15288602

[2] Green SR, Al-Attar R, McKechnie AE, Naidoo S, Storey KB. 2020 Role of Akt signaling pathway regulation in the speckled mousebird (*Colius striatus*) during torpor displays tissue specific responses. Cell. Signal. **70**, 109763. (10.1016/j.cellsig.2020.109763) 32871209

[3] Prinzinger R, Preßmar A, Schleucher E. 1991 Body temperature in birds. Comp. Biochem. Physiol. **99A**, 499-506. (10.1016/0300-9629(91)90122-S)

[4] Geiser F, Ruf T. 1995 Hibernation versus daily torpor in mammals and birds: physiological variables and classification of torpor patterns. Physiol. Zool. **68**, 935-966. (10.1086/physzool.68.6.30163788)

[5] McKechnie AE, Lovegrove BG. 2002 Avian facultative hypothermic responses: a review. Condor **104**, 705-724. (10.1093/condor/104.4.705)

[6] Ruf T, Geiser F. 2015 Daily torpor and hibernation in birds and mammals. Biol. Rev. **90**, 891-926. (10.1111/brv.12137) 25123049 PMC4351926

[7] Geiser F. 2004 Metabolic rate and body temperature reduction during hibernation and daily torpor. Annu. Rev. Physiol. **66**, 239-274. (10.1146/annurev.physiol.66.032102.115105) 14977403

[8] Geiser F. 2019 Frequent nocturnal torpor in a free-ranging Australian honeyeater, the noisy miner. Sci. Nat. **106**, 28. (10.1007/s00114-019-1626-9) 31134403

[9] Geiser F. 2020 Seasonal expression of avian and mammalian daily torpor and hibernation: not a simple summer-winter affair. Front. Physiol. **11**, 436. (10.3389/fphys.2020.00436) 32508673 PMC7251182

[10] Wolf BO, McKechnie AE, Schmitt CJ, Czenze ZJ, Johnson AB, Witt CC. 2020 Extreme and variable torpor among high-elevation Andean hummingbird species. Biol. Lett. **16**, 20200428. (10.1098/rsbl.2020.0428) 32898456 PMC7532710

[11] Geiser F, Brigham RM. 2012 The other functions of torpor. In Living in a seasonal world (eds T Ruf, C Bieber, W Arnold, E Millesi), pp. 109-122. Berlin, Germany: Springer.

[12] Nowack J, Stawski C, Geiser F. 2017 More functions of torpor and their roles in a changing world. J. Comp. Physiol. B **187**, 889-897. (10.1007/s00360-017-1100-y) 28432393 PMC5486538

[13] Doucette LI, Brigham RM, Pavey CR, Geiser F. 2012 Prey availability affects daily torpor by free-ranging Australian owlet-nightjars (*Aegotheles cristatus*). Oecologia **169**, 361-372. (10.1007/s00442-011-2214-7) 22173484

[14] Hohtola E. 2012 Thermoregulatory adaptations to starvation in birds. In Comparative physiology of fasting, starvation, and food limitation (ed. MD McCue), pp. 155-170. Berlin, Germany: Springer.

[15] McKechnie AE, Lovegrove BG. 2001 Thermoregulation and the energetic significance of clustering behavior in the white-backed mousebird (*Colius colius*). Physiol. Biochem. Zool. **74**, 238-249. (10.1086/319669) 11247743

[16] McKechnie AE, Lovegrove BG. 2001 Heterothermic responses in the speckled mousebird (*Colius striatus*). J. Comp. Physiol. B **171**, 507-518. (doi:0.1007/s003600100201) 11585263 10.1007/s003600100201

[17] McNab BK, Weston KA. 2018 The energetics of torpor in a temperate passerine endemic to New Zealand, the rifleman (*Acanthisitta chloris*). J. Comp. Physiol. B **188**, 855-862. (10.1007/s00360-018-1175-0) 30039298

[18] McNab BK, Weston KA. 2020 Does the New Zealand rockwren (*Xenicus gilviventris*) hibernate? J. Exp. Biol. **223**, jeb212126. (10.1242/jeb.212126) 32291323

[19] Fletcher QE, Fisher RJ, Willis CKR, Brigham RM. 2004 Free-ranging common nighthawks use torpor. J. Therm. Biol. **29**, 9-14. (10.1016/j.jtherbio.2003.11.004)

[20] Lane JE, Brigham RM, Swanson DL. 2004 Daily torpor in free-ranging whip-poor-wills (*Caprimulgus vociferus*). Physiol. Biochem. Zool. **77**, 297-304. (10.1086/380210) 15095249

[21] McAllan BM, Geiser F. 2014 Torpor during reproduction in mammals and birds: dealing with an energetic conundrum. Integr. Comp. Biol. **54**, 516-532. (10.1093/icb/icu093) 24973362

[22] Peiponen VA. 1965 On hypothermia and torpidity in the nightjar (*Caprimulgus europaeus* L). Ann. Acad. Sci. Fenn. Biol. **87**, 1-15.

[23] Bize P, Klopfenstein A, Jeanneret C, Roulin A. 2007 Intra-individual variation in body temperature and pectoral muscle size in nestling Alpine swifts *Apus melba* in response to an episode of inclement weather. J. Ornithol. **148**, 387-393. (10.1007/s10336-007-0141-5)

[24] Åkesson S, Klaassen R, Holmgren J, Fox JW, Hedenström A. 2012 Migration routes and strategies in a highly aerial migrant, the common swift *Apus apus*, revealed by light-level geolocators. PLoS ONE **7**, e41195. (10.1371/journal.pone.0041195) 22815968 PMC3399846

[25] Wellbrock AHJ, Bauch C, Rozman J, Witte K. 2017 ‘Same procedure as last year?‘ Repeatedly tracked swifts show individual consistency in migration pattern in successive years. J. Avian Biol. **48**, 897-903. (10.1111/jav.01251)

[26] Åkesson S et al. 2020 Evolution of chain migration in an aerial insectivorous bird, the common swift *Apus apus*. Evolution **74**, 2377-2391. (10.1111/evo.14093) 32885859 PMC7589357

[27] Lack D. 1956 Swifts in a tower. London, UK: Methuen & Co Ltd.

[28] Weitnauer E. 1980 Mein Vogel – Aus dem Leben des Mauerseglers Apus apus. Liestal, Switzerland: Basellandschaftlicher Natur- und Vogelschutzverband.

[29] Koskimies J. 1948 On temperature regulation and metabolism in the swift, *Micropus apus* L., during fasting. Experientia **4**, 274-276. (10.1007/BF02164408) 18875260

[30] Koskimies J. 1950 The life of the swift, *Micropus* *apus* (L.), in relation to the weather. Ann. Acad. Sci. Fenn. **A IV**, 1-151.

[31] Koskimies J. 1961 Fakultative Kältelethargie beim Mauersegler (*Apus apus*) im Spätherbst [in German with English summary]. Vogelwarte **22**, 161-166.

[32] Keskpaik J. 1973 Ontogenetic development of torpid cycle in the European swifts (*Apus a. apus* L.) [in Russian with English summary]. Eesti Nsv Tead. Akad. TOIM Biol. **22**, 113-121.

[33] Willis CKR, Goldzieher A, Geiser F. 2005 A non-invasive method for quantifying patterns of torpor and activity under semi-natural conditions. J. Therm. Biol. **30**, 551-556. (10.1016/j.jtherbio.2005.07.001)

[34] Weidinger K. 2006 Validating the use of temperature data loggers to measure survival of songbird nests. J. Field Ornithol. **77**, 357-364. (10.1111/j.1557-9263.2006.00063.x)

[35] Hartmann CA, Oring LW. 2006 An inexpensive method for remotely monitoring nest activity. J. Field Ornithol. **77**, 418-424. (10.1111/j.1557-9263.2006.00073.x)

[36] Moore T, de Tores P, Fleming PA. 2010 Detecting, but not affecting, nest-box occupancy. Wildl. Res. **37**, 240-248. (10.1071/WR09111)

[37] Cervencl A, Esser W, Maier M, Oberdiek N, Thyen S, Wellbrock A, Exo KM. 2011 Can differences in incubation patterns of common redshanks *Tringa totanus* be explained by variations in predation risk? J. Ornithol. **152**, 1033-1043. (10.1007/s10336-011-0696-z)

[38] Barclay RMR, Lausen CL, Hollis L. 2001 What's hot and what's not: defining torpor in free-ranging birds and mammals. Can. J. Zool. **79**, 1885-1890. (10.1139/z01-138)

[39] Dausmann KH, Glos J, Ganzhorn JU, Heldmaier G. 2004 Hibernation in a tropical primate. Nature **429**, 825-826. (10.1038/429825a) 15215852

[40] Canale CI, Levesque DL, Lovegrove BG. 2012 Tropical heterothermy: does the exception prove the rule or force a re-definition? In Living in a seasonal world (eds T Ruf, C Bieber, W Arnold, E Millesi), pp. 29-40. Berlin, Germany: Springer.

[41] PhenoSys. 2020 Indirect Calorimetry. CaloBox. See https://www.phenosys.com/wp-content/uploads/2019/10/PhenoSys_Brochure_CaloBox_1910.pdf.

[42] Schaub T, Wellbrock AHJ, Rozman J, Witte K. 2019 Light data from geolocation reveal patterns of nest visit frequency and suitable conditions for efficient nest site monitoring in common swifts *Apus apus*. Bird Study **66**, 519-530. (10.1080/00063657.2020.1732862)

[43] Spence AR, Tingle MW. 2021 Body size and environment influence both intraspecific and interspecific variation in daily torpor use across hummingbirds. Funct. Ecol. **35**, 870-883. (10.1111/1365-2435.13782)

[44] Bates D, Maechler M, Bolker B, Walker S. 2015 Fitting linear mixed-effects models using lme4. J. Stat. Softw. **67**, 1-48. (10.18637/jss.v067.i01)

[45] Lüdecke D. 2020 sjPlot: data visualization for statistics in social science. R package version 2.8.6. See https://CRAN.R-project.org/package=sjPlot.

[46] Korner-Nievergelt F, Roth T, von Felten S, Guélat J, Almasi B, Korner-Nievergelt P. 2015 Bayesian data analysis in ecology using linear models with R, BUGS, and Stan. Amsterdam, The Netherlands: Academic Press.

[47] R Development Core Team. 2020 R: a language and environment for statistical computing. Version. 4.0.3. Vienna, Austria: R Foundation for Statistical Computing.

[48] Jerem P, Jenni-Eiermann S, Herborn K, McKeegan D, McCaferty D, Nager R. 2018 Eye region surface temperature reflects both energy reserves and circulating glucocorticoids in a wild bird. Sci. Rep. **8**, 1907. (10.1038/s41598-018-20240-4) 29382942 PMC5789886

[49] Linek N, Volkmer T, Shipley JR, Twining CW, Zúñiga D, Wikelski M, Partecke J. 2021 A songbird adjusts its heart rate and body temperature in response to season and fluctuating daily conditions. Phil. Trans. R. Soc. B **376**, 20200213. (10.1098/rstb.2020.0213) 34121457 PMC8200648

[50] Vuarin P, Dammhahn M, Henry PY. 2013 Individual flexibility in energy saving: body size and condition constrain torpor use. Funct. Ecol. **27**, 793-799. (10.1111/1365-2435.12069)

[51] Shipley JR, Gu DY, Salzman TC, Winkler DW. 2015 Heterothermic flexibility allows energetic savings in a small tropical swift: the silver-rumped spinetail (*Rhaphidura leucopygialis*). Auk **132**, 697-703. (10.1642/AUK-15-15.1)

[52] McKechnie AE, Körtner G, Lovegrove BG. 2004 Rest-phase thermoregulation in free-ranging white-backed mousebirds. Condor **106**, 144-150. (10.1093/condor/106.1.143)

[53] McKechnie AE, Körtner G, Lovegrove BG. 2006 Thermoregulation under semi-natural conditions in speckled mousebirds: the role of communal roosting. Afr. Zool. **41**, 155-163. (10.1080/15627020.2006.11407350)

[54] Lovegrove BG, Smith GA. 2003 Is ‘nocturnal hypothermia' a valid physiological concept in small birds?: a study on bronze mannikins *Spermestes cucullatus*. Ibis **145**, 547-557. (10.1046/j.1474-919X.2003.00166.x)

[55] Nowack J, Geiser F. 2016 Friends with benefits: the role of huddling in mixed groups of torpid and normothermic animals. J. Exp. Biol. **219**, 590-596. (10.1242/jeb.128926) 26685170

[56] Jastroch M, Giroud S, Barrett P, Geiser F, Heldmaier G, Herwig A. 2016 Seasonal control of mammalian energy balance: recent advances in the understanding of daily torpor and hibernation. J. Neuroendocrinol. **28**, e.12437. (10.1111/jne.12437) 27755687

[57] Legendre LJ, Davesne D. 2020 The evolution of mechanisms involved in vertebrate endothermy. Phil. Trans. R. Soc. B **375**, 20190136. (10.1098/rstb.2019.0136) 31928191 PMC7017440

[58] Bicudo JEPW, Bianco AC, Vianna CR. 2002 Adaptive thermogenesis in hummingbirds. J. Exp. Biol. **205**, 2267-2273. (10.1242/jeb.205.15.2267) 12110660

[59] Nowack J, Giroud S, Arnold W, Ruf T. 2017 Muscle non-shivering thermogenesis and its role in the evolution of endothermy. Front. Physiol. **8**, 889. (10.3389/fphys.2017.00889) 29170642 PMC5684175

[60] Bal NC, Periasamy M. 2020 Uncoupling of sarcoendoplasmic reticulum calcium ATPase pump activity by sarcolipin as the basis for muscle non-shivering thermogenesis. Phil. Trans. R. Soc. B **375**, 20190135. (10.1098/rstb.2019.0135) 31928193 PMC7017432

[61] Grüebler MU, Morand M, Naef-Daenzer B. 2008 A predictive model of the density of airborne insects in agricultural environments. Agric. Ecosyst. Environ. **123**, 75-80. (10.1016/j.agee.2007.05.001)

[62] Ruuskanen S, Hsu BY, Nord A. 2021 Endocrinology of thermoregulation in birds in a changing climate. Mol. Cell. Endocrinol. **519**, 111088. (10.1016/j.mce.2020.111088) 33227349

[63] Wellbrock AHJ, Eckhardt LRH, Kelsey NA, Heldmaier G, Rozman J, Witte K. 2022 Data from: Cool birds: first evidence of energy-saving nocturnal torpor in free-living common swifts *Apus apus* resting in their nests. *Dryad Digital Repository*. (10.5061/dryad.6wwpzgn1f) PMC9006018 35414223

[64] Wellbrock AHJ, Eckhardt LRH, Kelsey NA, Heldmaier G, Rozman J, Witte K. 2022 Cool birds: first evidence of energy-saving nocturnal torpor in free-living common swifts *Apus apus* resting in their nests. *FigShare*. (10.6084/m9.figshare.c.5918312) PMC9006018 35414223

